# A flat posterior cruciate ligament reconstruction restores native knee kinematics, comparable to a double‐bundle reconstruction—A biomechanical robotic investigation

**DOI:** 10.1002/ksa.12572

**Published:** 2024-12-30

**Authors:** Adrian Deichsel, Florian Gellhaus, Christian Peez, Michael J. Raschke, Moritz Martinovic, Elmar Herbst, Mirco Herbort, Christian Fink, Christoph Kittl

**Affiliations:** ^1^ Department of Trauma, Hand and Reconstructive Surgery University Hospital Münster Münster Germany; ^2^ Department of Anatomy Christian‐Albrechts‐University Kiel Germany; ^3^ OCM Clinic Munich Germany; ^4^ Research Unit for Orthopedic Sports Medicine and Injury Prevention (OSMI) UMIT Hall Austria; ^5^ Gelenkpunkt Sports and Joint Surgery Innsbruck Innsbruck Austria

**Keywords:** biomechanics, posterior cruciate ligament, PCL, reconstruction, rectangular

## Abstract

**Purpose:**

To biomechanically evaluate a flat posterior cruciate ligament (PCL) reconstruction utilizing rectangular femoral bone tunnels.

**Methods:**

Eight fresh‐frozen human knee specimens were tested in a six‐degrees‐of‐freedom robotic test setup. In each testing step, a force‐controlled test protocol was performed, including 89 N posterior tibial translation (PTT) in neutral, internal and external rotation, from 0 to 90° of flexion. After determining the native knee kinematics, the PCL was cut. Subsequently, a flat PCL reconstruction (PCLR) with a rectangular bone tunnel was performed, utilizing a quadriceps tendon autograft with a patellar bone block. After filling the bone tunnel, a single‐bundle PCLR without and with femoral interference screw fixation, as well as a double‐bundle reconstruction, was performed. Statistical analysis was performed using mixed linear models.

**Results:**

Cutting of the PCL led to significant (*p *≤ .05) increases in PTT, from 0 to 90° of flexion, up to 10.7 mm, in comparison to the native state. After flat reconstruction and double‐bundle reconstruction, no significant difference was found between the native and reconstructed state (*p *≥ .05). The single‐bundle PCLR without interference screw showed significantly increased PTT in comparison to the native state in 30° (mean difference [MD] 3.3 mm; 95% confidence interval [CI] 1.3 – 5.2 mm; *p *< .001), 60° (MD 4.4 mm; 95% CI 2.5–6.4 mm; *p *< .001) and 90° of flexion (MD 4.0 mm; 95% CI 2.1–6.0 mm; *p *< .001). The single‐bundle PCLR with additional interference screw showed significantly increased PTT in comparison to the native state only in 30° (MD 1.9 mm; 95% CI 0.05–3.8 mm; *p *= .01).

**Conclusion:**

Both a flat and a double‐bundle PCLR were able to restore the native knee kinematics in all tested flexion angles. A single‐bundle reconstruction was not able to fully restore native kinematics, with only small residual anteroposterior instability.

**Abbreviations**
ACL: anterior cruciate ligament
ALB: anterolateral bundle
CI: confidence interval
ER: external rotation
IR: internal rotation
MD: mean difference
PCL: posterior cruciate ligament
PCLR: posterior cruciate ligament reconstruction
PMB: posteromedial bundle
PTT: posterior tibial translation

## INTRODUCTION

To restore the native function of the posterior cruciate ligament (PCL)‐deficient knee as closely as possible, a PCL reconstruction (PCLR) imitating the native restraint is strived for [35, 36]. Varying anatomical descriptions of the PCL exist in the literature, ranging from the most frequently used double‐bundle structure (consisting of the anterolateral bundle [ALB] and the posteromedial bundle [PMB]) to flat, ribbon‐like ligaments [11, 23, 26]. Therefore, the optimal technique for PCLR is still debated [4, 25, 32, 36, 43]. Mostly, a single‐bundle PCLR, reconstructing the ALB, is performed using either autologous or allogenous tendon grafts [43, 44]. Double‐bundle PCLR of both the ALB and PMB were shown to be biomechanically superior to single‐bundle PCLR but are technically more demanding and require the use of multiple tendons [36, 41]. Furthermore, the clinical superiority of the double‐bundle technique could not be established [44].

In a previous biomechanical study, a rectangular area towards the proximal and posterior part of the femoral PCL footprint was found to be the main contributor to the PCLs ability to restrain a posterior tibial drawer [7]. Incorporating these findings, an adapted technique for PCLR, utilizing a rectangular femoral bone tunnel, which allows reconstruction of the aforementioned areas, was developed [14]. The purpose of this study was to biomechanically evaluate this new technique and to compare it to established single‐ and double‐bundle PCLR techniques. It was hypothesized that the novel technique for PCLR would restore the physiological knee kinematics, with comparable results to a double‐bundle technique. It was furthermore hypothesized that a single‐bundle reconstruction of the PCL would not be able to fully restore the native knee kinematics.

## MATERIALS AND METHODS

Eight unpaired cadaveric knee specimens (mean age 65 years, range 45–80 years, five female, three male, four left, four right) without a history of prior knee surgery or injury were obtained from an international tissue bank (MedCure, Portland, USA). The specimens were visually inspected for high‐grade osteoarthritis (Kellgren–Lawrence score > 2) or meniscal injury. Furthermore, ligamentous instability testing was performed by the investigator. Specimens were excluded if pathologies were present.

Specimens were stored at –20°C and thawed for 24 h at room temperature before preparation. After resecting skin and subcutaneous tissue, the tibia and femur were secured in aluminium cylinders. This was performed 12 cm above and below the joint line with three‐component polyurethane bone cement (RenCast®; Gößl & Pfaff). The fibula was then cut 10 cm below the joint line and transfixed with a 3.5 mm cortical screw to the tibia [38]. Specimens were regularly sprayed with water and wrapped in moist tissue papers to prevent drying [45].

### Robotic test setup

A validated test setup consisting of a six‐degrees‐of‐freedom industrial robot (KR 60‐3; KUKA Robotics) equipped with a force‐torque sensor (FTI Theta; ATI Industrial Automation, Apex) was used for biomechanical testing in this study, as described in previous studies [7, 8, 9]. The robot allows for position‐controlled movement with an accuracy of ±0.06 mm and force‐controlled movement with an accuracy of ±0.25 N and ±0.05 Nm, respectively (test–retest reliability). The test setup was optimized for the simulation of knee joint movements by the custom software simVITRO (Cleveland Clinic BioRobotics Lab). A modified Grood and Suntay coordinate system was created for each specimen by digitizing landmarks on the femur and tibia with a tactile measuring arm (Absolute Arm 8320‐7; Hexagon Metrology), which has an accuracy of ±0.05 mm. The sampling rate of the test setup was set to 500 Hz.

### Biomechanical testing

Tissue hysteresis was obtained by flexing and extending each specimen 10 times [28]. The starting point of each knee was determined by manually minimizing all forces and torques acting on the knee in full extension. The passive path of the knee was then determined by moving each knee from full 0–90° of flexion while minimizing forces and torques in all axes aside from the flexion‐extension axis. During the identification of the passive path, an axial compression force of 50 N was applied to keep contact between the femur and tibia. In the following, a force‐controlled test protocol (recording displacements in response to given forces/torques) was performed under 200 N of axial compression (to simulate partial weightbearing) in 0°, 30°, 60° and 90° of flexion: 89 N posterior tibial translation (PTT) force (simulating the forces applied by the Telos device (Telos Medical) during a posterior drawer [18]), 89 N PTT in 5 Nm internal rotation (IR; simulating a posteromedial drawer test [37, 42]) and 89 N PTT in 5 Nm external rotation (ER; simulating a posterolateral drawer test [16, 21]).

### Sequential cutting protocol

Before the evaluation of the native state, a longitudinal transpatellar osteotomy was performed according to the descriptions of Merican et al. to gain access to the central compartment for resection of the PCL and drilling of the femoral tunnels [29]. The osteotomy was performed at the medial border of the quadriceps to allow harvesting of the tendon graft for later reconstruction. The osteotomy was closed with two 3.5 mm cortical screws, which allowed repeated opening and closing of the osteotomy. Furthermore, a posterior approach to the tibial footprint of the PCL was established by horizontally splitting the posterior joint capsule at the centre of the tibial width, between the oblique popliteal ligament and the superior border of the popliteus muscle. After establishment of the approach, the native knee kinematics were determined, using the force‐controlled test protocol. Using blunt preparation, as previously described [3, 15], the ALB and PMB were identified and cut in a randomized order. Subsequently, reconstructions of the PCL were performed in the following order: (1) Flat PCLR, using a rectangular femoral bone tunnel; (2) Single‐bundle ALB reconstruction with femoral fixation with a cortical button; (3) Single‐bundle ALB reconstruction with additional femoral fixation by an interference screw; (4) Double‐bundle ALB and PMB reconstruction. Due to the ways the autografts were used in the present study, randomization of the reconstructive techniques was not feasible.

### Surgical technique of flat PCLR

**Figure 1:**
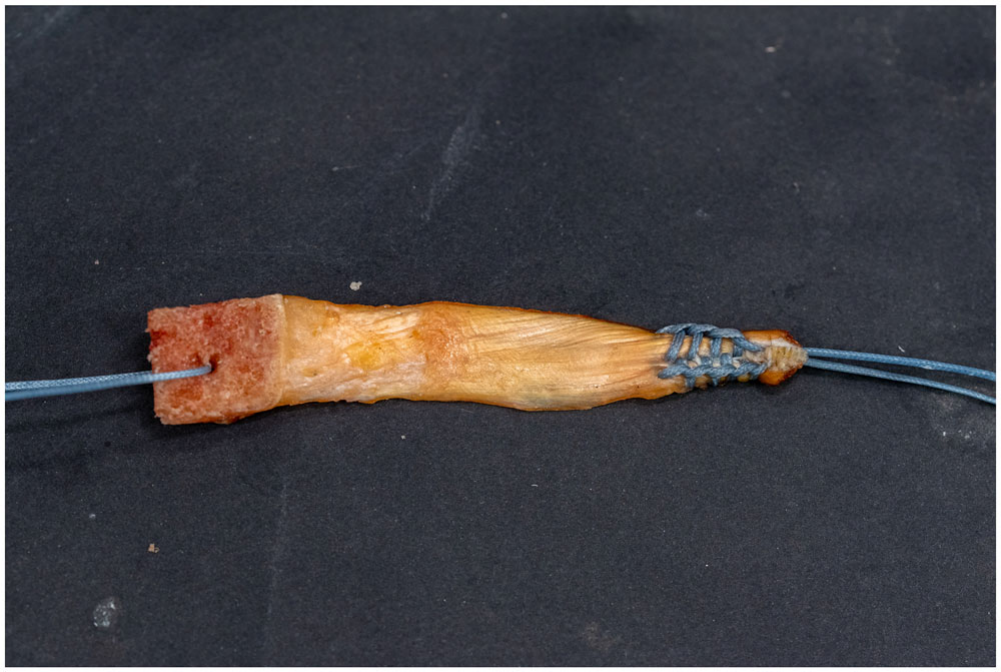
Quadriceps autograft with patellar bone block for flat posterior cruciate ligament reconstruction.

A 14 × 4 mm thick strip of the superficial layer of the quadriceps tendon (8 cm length) was extracted, with a patellar bone block of corresponding size (Figure 1), utilizing an oscillating saw (DePuy Synthes). A hole was drilled through the bone block by use of a 2.0 mm Kirschner‐wire (K‐wire). A high‐strength polyethylene suture (No. 5 FiberWire, Arthrex) was passed through the hole to facilitate pull‐in of the bone block into the femoral tunnel. The size of the bone block was confirmed using a special sizing block for rectangular grafts, originally designed for anterior cruciate ligament (ACL) reconstruction (Medacta). The tibial end was sutured with the same suture material in Krackow technique [30]. The diameter of the tibial graft end was measured using a standard ACL graft sizing instrument (Karl Storz).

**Figure 2:**
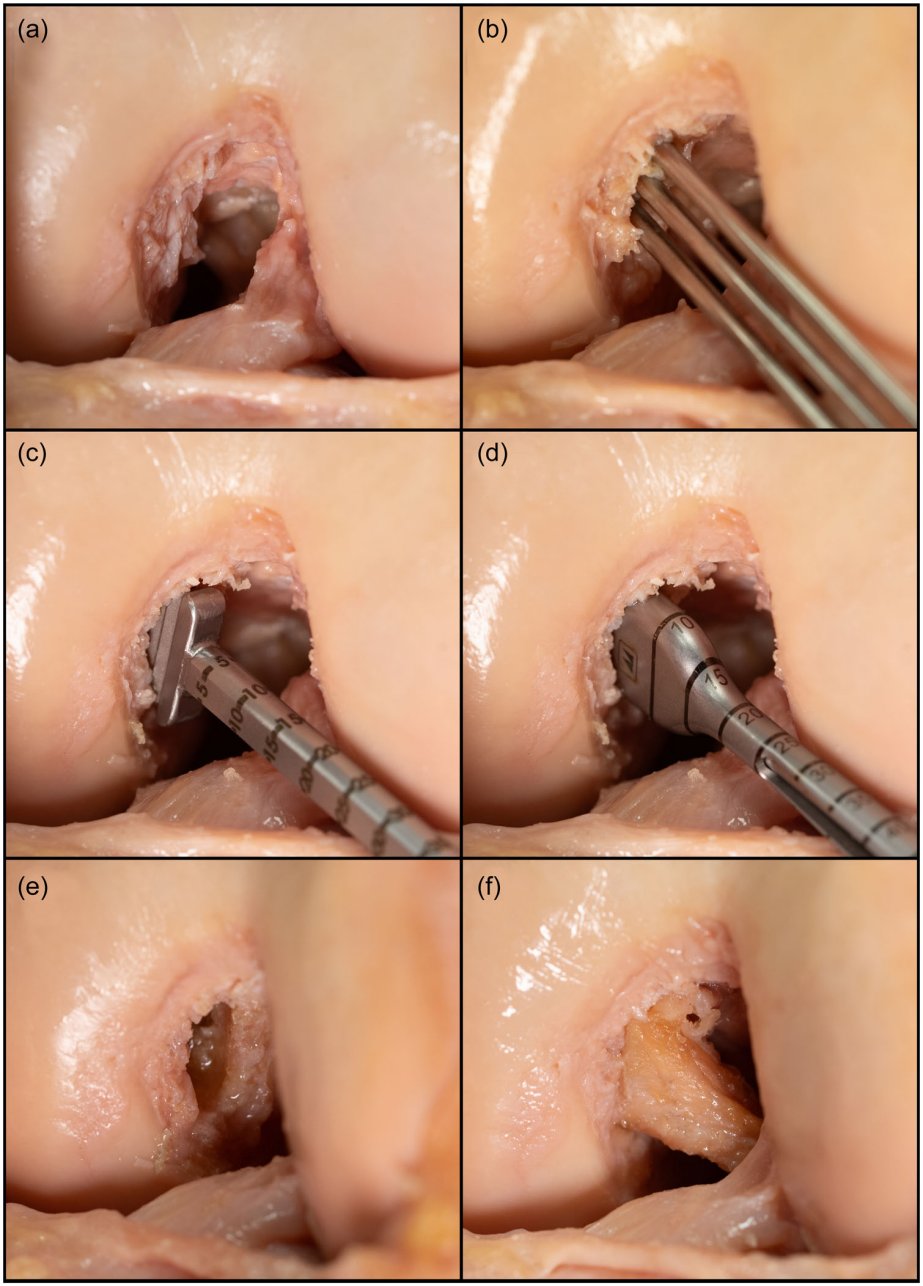
Technique of flat posterior cruciate ligament (PCL) reconstruction in a right knee. After resection of the PCL (a), three 2.0 mm Kirschner‐wires were placed parallel to the medial intercondylar ridge (b) and subsequently overdrilled with a 4.5 mm cannulated drill. The created oval tunnel was enlarged with a cannulated rasp (c) and cannulated dilator (d) to create a rectangular bone tunnel (e), into which a quadriceps autograft with patella bone block was inserted (f).

For the preparation of the femoral tunnel, special instruments for the creation of rectangular tunnels were used (Medacta Anatomic Ribbon Surgery; M‐ARS), similar to a previously described technique [14]. After resection of the PCL from the femoral and tibial footprint, three 2.0 mm K‐wires were placed slightly in front of the medial intercondylar ridge (Figure 2) using an aiming device. The K‐wires were overdrilled with a 4.5 mm cannulated drill. Subsequently, the tunnel was enlarged to the appropriate size by using cannulated rasps and dilators. The tibial tunnel was created by placing a K‐wire centrally into the tibial footprint of the PCL. After overdrilling with a 4.5 mm cannulated drill, the tunnel was enlarged to the appropriate size with the corresponding cannulated drill. The bone block was pulled into the femoral tunnel, and femoral graft fixation was performed with a cortical button (FlippTack; Karl Storz). Fixation of the tibial side was performed utilizing a custom‐made tensioning device [33]. The device allows tensioning and measurement of the forces acting on the tibial graft sutures. The sutures were connected to a load cell (Y1 in‐line threaded force transducer, Flintec GmbH), which was connected over an amplifier (DAD141.1, Flintec GmbH) to a data acquisition device (USB 6343, National Instruments), connected to a laptop running a custom‐made LabView (LabVIEW 2020, National Instrument) script for data acquisition. Utilizing the tensioning device, graft fixation was performed with 60 N at 90° of flexion [2, 27]. After tensioning of the PCLR, each knee was moved through the full range of motion, with subsequent re‐tensioning, to achieve preconditioning of the construct [22].

### Surgical technique of single‐ and double‐bundle reconstruction

**Figure 3:**
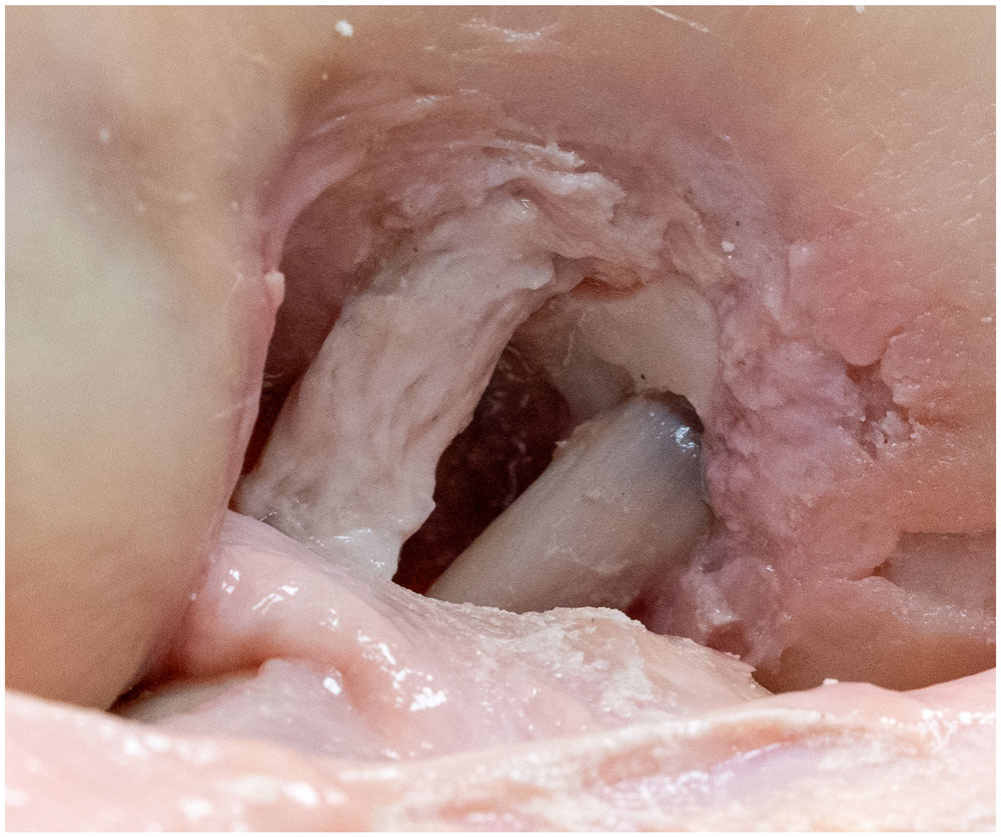
Medial notch wall after insertion of a double‐bundle posterior cruciate ligament reconstruction, in a left knee, with a quadriceps tendon autograft in the anterolateral position and semitendinosus tendon autograft in the posteromedial position.

After extraction of the patellar bone block from the femoral tunnel, the residual hole was filled with three‐component polyurethane bone cement (RenCast®; Gößl & Pfaff). The bone block was cut from the former flat graft, and the femoral side of the quadriceps tendon was sutured in the Krackow technique to create a round ALB graft for the following reconstructions. This ensured the comparability of the grafts between the different PCLR techniques. The diameter of the graft was measured, and a 2.0 mm K‐wire was inserted in the centre of the ALB, followed by sequential overdrilling with a 4.5 mm cannulated drill. A bone socket was then created corresponding to the graft diameter (mean 9.4 ± 1 mm, range 8–11 mm). The graft was pulled into the tunnel, and femoral graft fixation was performed with a cortical button (FlippTack; Karl Storz). Tibial fixation was performed exactly as in the flat reconstruction. After testing the single‐bundle ALB reconstruction with cortical button fixation, an additional femoral interference (FastThread™ biocomposite, Arthrex) of 1 mm less diameter than the tunnel was positioned behind the graft, as previously described [34, 40]. After testing the single‐bundle ALB reconstruction with additional interference screw, double‐bundle reconstruction was performed utilizing a doubled autologous semitendinosus graft (diameter approximately 4.5 mm) from the same knee, in addition to the previously placed single‐bundle reconstruction in an ALB position (Figure 3). The femoral PMB tunnel was placed, as previously described [6]. After pulling the graft into the tunnel, it was fixed with an interference screw, analogous to the ALB. On the tibial side, the PMB was fixed together with the ALB graft in the same tunnel. Graft fixation for the single‐bundle reconstructions, as well as the ALB in double‐bundle reconstruction, was performed with 60 N at 90° of flexion. The PMB was fixed with 60 N in full extension [2, 6].

### Institutional review board approval

The study was approved by the institutional review board of the University of Münster (IRB reference number 2023‐407‐f‐S).

### Data analysis, statistics and sample size calculation

Extraction of knee kinematics from the raw data of SimVitro was performed using Matlab (version R2020a, MathWorks) and Excel (Microsoft). Statistical analysis was performed using PRISM (version 10, GraphPad Software). The influence of the cutting and reconstruction states on the PTT was analyzed utilizing mixed linear models. Post hoc pairwise comparisons with Dunnet correction were used to investigate the influence of each cutting state. Comparisons were performed against the native state to refrain from unnecessary multiple comparisons. Furthermore, the single‐bundle reconstruction with and without interference screw and the flat and double‐bundle reconstruction were compared with pairwise comparisons. A *p *< 0.05 was deemed to identify significant differences. Results are presented as means ± standard deviations (SD). Between‐group differences are presented as mean differences (MD) with corresponding 95% confidence intervals (CI).

To calculate the sample size necessary for the present study, an a priori power analysis was performed using G*Power (version 3.1, HHU Düsseldorf) [12]. Based on a previous study investigating the influence of different reconstruction techniques of the PCL [41], a sample size of *n* = 8 was calculated to show a 3 mm between‐group difference (assuming an SD of 2 mm; Cohens *d* = 1.5), with a power of 95%, at the significance level of *p *< .05.

## RESULTS

No specimen had to be excluded, resulting in every tested knee being available for final analysis.

### Instability caused by cutting of the PCL

**Figure 4:**
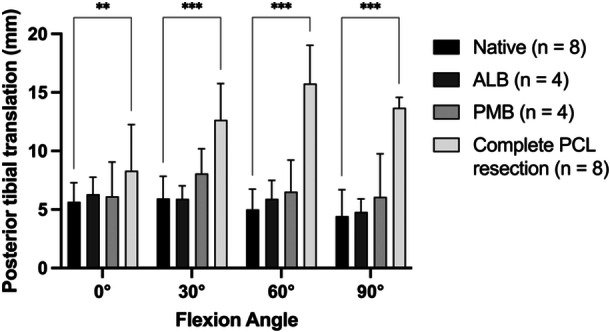
Instability caused by randomized sequential cutting of the anterolateral bundle (ALB) and posteromedial bundle (PMB) and complete resection of the posterior cruciate ligament (PCL). *p < .05; **p < .01; ***p < .001.

PTT in the native state was 5.7 ± 1.6 mm in 0° of flexion, 5.9 ± 1.9 mm in 30° of flexion, 5.0 ± 1.7 mm in 60° of flexion and 4.4 ± 2.6 mm in 90° of flexion. Neither cutting the ALB first (*n *= 4) nor PMB first (*n *= 4) led to a significant increase in PTT in comparison to the native state (Figure 4). Full deficiency led to a significant increase in 0° (MD 2.6 mm; 95% CI 0.7–4.5 mm; *p* = .002), 30° (MD 6.7 mm; 95% CI 4.8–8.6 mm; *p *< .001), 60° (MD 10.7 mm; 95% CI 8.9–12.6 mm; *p *< .001) and 90° of flexion (MD 9.3 mm; 95% CI 7.4–11.1 mm; *p *< .001).

### Effect of reconstruction on PTT

**Figure 5:**
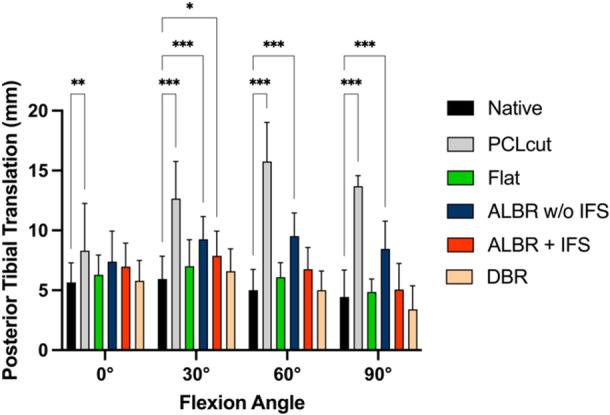
Influence of cutting the posterior cruciate ligament (PCL) with subsequent reconstructions on posterior tibial translation. ALBR, anterolateral bundle reconstruction; DBR, double‐bundle reconstruction; IFS, interference screw; Multiple comparisons were performed against the native state. *p< .05; **p < .01; ***p < .001.

After flat reconstruction of the PCL, no significant difference was found between the native and reconstructed state (*p *≥ .05; Figure 5). The single‐bundle PCLR without interference screw showed significantly increased PTT in comparison to the native state in 30° (MD 3.3 mm; 95% CI 1.3–5.2 mm; *p *< .001), 60° (MD 4.4 mm; 95% CI 2.5–6.4 mm; *p *< .001) and 90° of flexion (MD 4.0 mm; 95% CI 2.1–6.0 mm; *p *< .001). The single‐bundle PCLR with additional interference screw showed significantly increased PTT in comparison to the native state in 30° (MD 1.9 mm; 95% CI 0.05–3.8 mm; *p *= .01). No significant difference was found between the native state and the double‐bundle PCLR (*p *≥ .05).

In the pairwise comparisons, the single‐bundle reconstruction with additional femoral interference screw fixation showed significantly less PTT in 30° (MD 1.3 mm; 95% CI 0.1–2.6 mm; *p *< .01), 60° (MD 2.6 mm; 95% CI 0.5–4.7 mm; *p *= .02) and 90° of flexion (MD 3.5 mm; 95% CI 0.1–6.9 mm; *p *= .04) in comparison to the single‐bundle reconstruction with only cortical button fixation. A pairwise comparison of the flat reconstruction and double‐bundle reconstruction revealed no significant differences between the groups (*p *≥ .05).

### Effect of cutting and reconstruction on PTT in rotation

**Figure 6:**
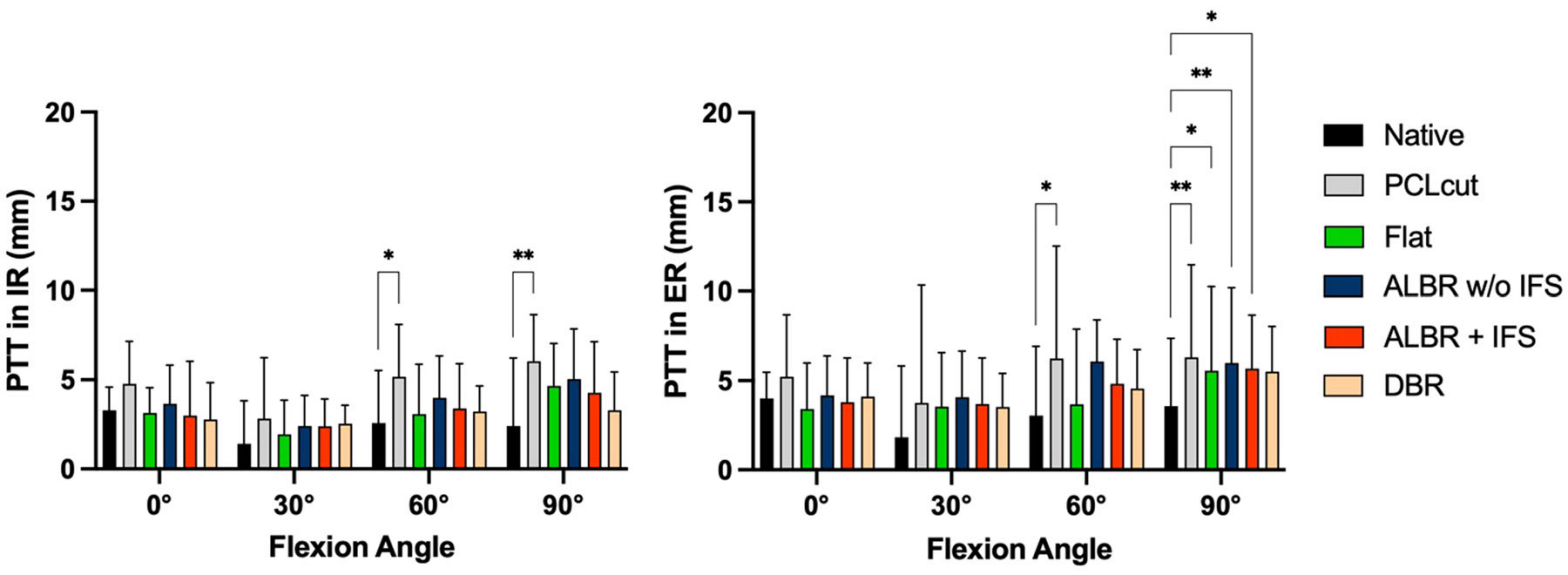
Influence of cutting the posterior cruciate ligament (PCL) with subsequent reconstructions on posterior tibial translation (PTT) in internal rotation (IR) and external rotation (ER). ALBR, anterolateral bundle reconstruction; DBR, double‐bundle reconstruction; IFS, interference screw; Multiple comparisons were performed against the native state; *p < .05; **p < .01; ***p < .001.

PTT in IR (posteromedial drawer), in the native state, was 3.3 ± 1.3 mm in 0° of flexion, 1.4 ± 2.4 mm in 30° of flexion, 2.6 ± 2.9 mm in 60° of flexion and 2.4 ± 3.8 mm in 90° of flexion. A deficiency of the PCL led to a significant increase of PTT in 60° (MD 2.6 mm; 95% CI 0.3–3.5 mm; *p *= .02) and 90° of flexion (MD 3.6 mm; 95% CI 1.1–6.1 mm; *p *= .009). After reconstruction with any of the techniques described, no significant difference between the reconstructed state and the native state was found in any of the tested flexion angles (Figure 6; *p *≥ .05).

PTT in ER (posterolateral drawer), in the native state, was 4.0 ± 1.5 mm in 0° of flexion, 1.8 ± 4.0 mm in 30° of flexion, 3.0 ± 3.8 mm in 60° of flexion and 3.6 ± 3.8 mm in 90° of flexion. A deficiency of the PCL led to a significant increase of PTT in 60° (MD 3.2 mm; 95% CI 0.1–6.3 mm; *p *= .04) and 90° of flexion (MD 2.7 mm; 95% CI 0.8–4.6 mm; *p *= .008). In 90° of flexion, the flat reconstruction (MD 1.9 mm; 95% CI 0.4–3.6 mm; *p *= .02) as well as the single‐bundle reconstruction with (MD 2.1 mm; 95% CI 0.6–3.6 mm; *p *= .01) and without interference screw (MD 2.4 mm; 95% CI 0.7–4.1 mm; *p *= .009) showed significantly increased PTT values, in comparison to the native state.

## DISCUSSION

The most important finding of the present study was that both a flat reconstruction of the PCL and a double‐bundle reconstruction restored the native knee kinematics in the PCL‐deficient knee in this biomechanical time‐zero study. A single‐bundle reconstruction of the ALB, with femoral interference screw fixation, was able to restore the native knee kinematics in 0°, 60° and 90° of flexion, with small residual instability (~2 mm) in 30° of flexion, which is of unclear clinical relevance. A single‐bundle reconstruction utilizing only cortical button fixation on the femoral side showed significantly inferior results in comparison to the single‐bundle reconstruction utilizing femoral interference screw fixation from 30 to 90° of flexion.

Previously, several biomechanical studies investigated the optimal reconstruction of the PCL. Although multiple anatomical variants are described, the PCL is typically described to be composed of two functional bundles, which are the ALB and PMB [3, 15]. When evaluating the physiological function of the PCL, it was shown by numerous studies that the ALB and PMB show reciprocal function and that both bundles have to be deficient to lead to clinically meaningful instability in the posterior direction [1, 24]. This is congruent to the present studies' results, showing that isolated cutting of either the ALB or PMB did not lead to significant increases in PTT. It was, therefore, postulated that a reconstruction of the PCL must aim to reconstruct as much of the two bundles as possible, which led to the development of a double‐bundle PCLR [13]. Indeed, in a landmark study performed on cadaveric knee specimens using a material testing machine, it was shown that neither a single‐bundle PCLR of the ALB nor an isometric PCLR, but only a double‐bundle PCLR, was able to fully restore the native knee kinematics [36]. A further biomechanical study used a robotic test system, similar to the one used in the present study, to compare single‐ and double‐bundle PCLR in cadaveric knee specimens [41]. This study found that a double‐bundle PCLR was able to restore the native knee kinematics ≥15° of flexion, while the single‐bundle PCLR in the ALB position showed significantly increased PTT. In comparison to the native state in all flexion angles, the difference in PTT ranged from 1.8 to 5.6 mm. These results are similar to the present study, which showed residual instability of a single‐bundle PCLR ranging from 3.3 mm in 30° of flexion to 4.0 mm in 90° of flexion in comparison to the native state. Interestingly, applying an additional femoral interference screw to the single‐bundle PCLR reduced residual instability to only 1.9 mm residual instability in 30° of flexion. This effect might be explained by the residual movement of the graft inside the tunnel when only an extracortical fixation is used. Although statistically significant, the difference between a single‐bundle PCLR with an interference screw and the native state was small and might not be clinically important or detectable [31].

Controversy exists regarding the optimal technique for PCLR [5, 17, 39, 43, 44]. Although biomechanical data suggested favourable results for certain surgical techniques (double‐bundle PCLR; [36, 41] tibial inlay techniques [4]), clinical superiority of such techniques could never be established [25, 44]. Based on the findings of the present study, from a biomechanical standpoint, a flat PCLR, double‐bundle PCLR and also a single‐bundle PCLR with additional femoral interference screw fixation provide restoration of the native knee kinematics in the PCL‐deficient knee. However, a flat reconstruction of the PCL might possess further distinct advantages. A rectangular femoral tunnel with a single tibial tunnel allows for less grafts to be used as well as an easier surgical technique in comparison to a double‐bundle reconstruction. Furthermore, a flat PCLR might lead to improved graft‐to‐bone healing, due to optimized tendon surface, inside the bone tunnel [46, 47].

Several limitations have to be considered when interpreting the results of the present study. As with most cadaveric biomechanical studies, knee specimens of older age were used for testing. In the present study, a quadriceps autograft with patellar bone block was used, as this technique was most reproducible in the cadaveric specimens. However, in clinical reality, harvesting a big quadriceps graft with patellar bone block might pose a risk for patellar fracture. Furthermore, arthroscopic insertion of the bone block into the femoral tunnel might be challenging. Alternatively, soft tissue grafts from both hamstring tendons and quadriceps tendons were previously shown to be adequate grafts for flat reconstructions as well [10, 14, 20]. For the tibial preparation, a single tibial tunnel in the centre of the PCL footprint was chosen, as this allowed the same fixation for all PCLR techniques performed. Different techniques exist for tibial tunnel placement during PCLR, with none of the techniques showing relevant biomechanical advantages [43, 44]. In the present study, no randomization of the different reconstructive techniques was applied, and femoral tunnels were filled with bone cement between different reconstructions, which could have possibly influenced the results, as the bone cement might be denser than the original bone. Finally, multiple PCLR techniques in the present study showed no significant difference to the native state. This, however, does not necessarily mean equivalence to the native state, as this study was only powered to test for differences between states, but underpowered to determine equivalence of states [19]. However, both flat and double‐bundle PCLR did not show a clear trend toward residual instability.

## CONCLUSION

A flat single‐bundle PCLR with rectangular femoral tunnel and a double‐bundle PCLR were able to restore the native knee kinematics in all tested flexion angles. A single‐bundle anterolateral reconstruction with interference screw was not able to restore the native knee kinematics in 30° of flexion with only small residual instability. Even though the proposed flat PCLR is of experimental nature, it provided similar restraint to PTT as the double‐bundle reconstruction and may be a future option for PCLRs using only a single graft. The proposed flat PCLR technique might lead to favourable clinical outcomes.

## AUTHOR CONTRIBUTIONS

**Adrian Deichsel**: Conception and design; testing and data acquisition; statistical analysis; writing; proofreading. **Florian Gellhaus**: Testing and data acquisition; internal review; proofreading. **Christian Peez**: Internal review; proofreading. **Michael J. Raschke**: Supervision; internal review; proofreading. **Moritz Martinovic**: Testing and data acquisition; proofreading. **Elmar Herbst**: Internal review; proofreading. **Mirco Herbort**: Internal review; proofreading. **Christian Fink**: Conception and design; internal review; proofreading. **Christoph Kittl**: Supervision; conception and design; statistical analysis; proofreading.

## CONFLICT OF INTEREST STATEMENT

Elmar Herbst is deputy editor‐in‐chief for the Knee Surgery, Sports Traumatology and Arthroscopy (KSSTA). Adrian Deichsel is the web editor for the Knee Surgery, Sports Traumatology and Arthroscopy (KSSTA). The remaining authors declare no conflict of interest.

## ETHICS STATEMENT

The specimens were dissected and biomechanically tested under the approval of the Institutional Ethics Committee of the University of Muenster (File number 2023‐407‐f‐S).

## Data Availability

Data are available from the corresponding author upon reasonable request.

## References

[1] Ahmad, C.S. , Cohen, Z.A. , Levine, W.N. , Gardner, T.R. , Ateshian, G.A. & Mow, V.C. (2003) Codominance of the individual posterior cruciate ligament bundles. An analysis of bundle lengths and orientation. The American Journal of Sports Medicine, 31, 221–225. Available from: 10.1177/03635465030310021101 12642256

[2] Amis, A.A. & Jakob, R.P. (1998) Anterior cruciate ligament graft positioning, tensioning and twisting. Knee Surgery, Sports Traumatology, Arthroscopy, 6(Suppl 1), S2–S12. Available from: 10.1007/s001670050215 9608456

[3] Anderson, C.J. , Ziegler, C.G. , Wijdicks, C.A. , Engebretsen, L. & LaPrade, R.F. (2012) Arthroscopically pertinent anatomy of the anterolateral and posteromedial bundles of the posterior cruciate ligament. Journal of Bone and Joint Surgery, 94, 1936–1945. Available from: 10.2106/JBJS.K.01710 23138236

[4] Berg, E.E. (1995) Posterior cruciate ligament tibial inlay reconstruction. Arthroscopy: The Journal of Arthroscopic & Related Surgery, 11, 69–76. Available from: 10.1016/0749-8063(95)90091-8 7727015

[5] Byrne, K.J. , Hughes, J.D. , Gibbs, C. , Vaswani, R. , Meredith, S.J. , Popchak, A. et al. (2022) Non‐anatomic tunnel position increases the risk of revision anterior cruciate ligament reconstruction. Knee Surgery, Sports Traumatology, Arthroscopy, 30, 1388–1395. Available from: 10.1007/s00167-021-06607-7 33983487

[6] Chahla, J. , Nitri, M. , Civitarese, D. , Dean, C.S. , Moulton, S.G. & LaPrade, R.F. (2016) Anatomic double‐bundle posterior cruciate ligament reconstruction. Arthroscopy Techniques, 5, e149–e156. Available from: 10.1016/j.eats.2015.10.014 27284530 PMC4886264

[7] Deichsel, A. , Briese, T. , Liu, W. , Raschke, M.J. , Albert, A. , Peez, C. et al. (2024) Specific fibre areas in the femoral footprint of the posterior cruciate ligament act as a major contributor in resisting posterior tibial displacement: a biomechanical robotic investigation. Knee Surgery, Sports Traumatology, Arthroscopy, n/a. Available from: 10.1002/ksa.12486 PMC12104786 39327853

[8] Deichsel, A. , Miets, H. , Peez, C. , Raschke, M.J. , Klimek, M. , Glasbrenner, J. et al. (2024) The effect of varying sizes of ramp lesions in the ACL‐deficient and reconstructed knee: a biomechanical robotic investigation. The American Journal of Sports Medicine, 52, 928–935. Available from: 10.1177/03635465231223686 38343294

[9] Deichsel, A. , Peez, C. , Raschke, M.J. , Albert, A. , Herbort, M. , Kittl, C. et al. (2024) A Flat reconstruction of the medial collateral ligament and anteromedial structures restores native knee kinematics: a biomechanical robotic investigation. The American Journal of Sports Medicine, 52, 3306–3313. Available from: 10.1177/03635465241280984 39360333 PMC11542325

[10] Domnick, C. , Herbort, M. , Raschke, M.J. , Schliemann, B. , Siebold, R. , Śmigielski, R. et al. (2017) Converting round tendons to flat tendon constructs: does the preparation process have an influence on the structural properties? Knee Surgery, Sports Traumatology, Arthroscopy, 25, 1561–1567. Available from: 10.1007/s00167-015-3749-7 26272060

[11] Edwards, A. , Bull, A.M.J. & Amis, A.A. (2007) The attachments of the fiber bundles of the posterior cruciate ligament: an anatomic study. Arthroscopy: The Journal of Arthroscopic & Related Surgery, 23, 284–290. Available from: 10.1016/j.arthro.2006.11.005 17349472

[12] Faul, F. , Erdfelder, E. , Buchner, A. & Lang, A.‐G. (2009) Statistical power analyses using G*Power 3.1: Tests for correlation and regression analyses. Behavior Research Methods, 41, 1149–1160. Available from: 10.3758/BRM.41.4.1149 19897823

[13] Finger, S. & Paulos, L. (2001) The history of posterior cruciate ligament injury and reconstruction. Techniques in Orthopaedics, 16, 105–108. Available from: 10.1097/00013611-200106000-00001

[14] Fink, C. , Farinelli, L. , Abermann, E. , Meena, A. , Smigielski, R. & Herbort, M. (2023) Posterior cruciate ligament reconstruction using flat soft‐tissue grafts. Arthroscopy Techniques, 12, e261–e271. Available from: 10.1016/j.eats.2022.10.016 36879862 PMC9984795

[15] Forsythe, B. , Harner, C. , Martins, C.A.Q. , Shen, W. , Lopes, Jr., O.V. , Fu, F.H. (2009) Topography of the femoral attachment of the posterior cruciate ligament. Surgical technique. Journal of Bone and Joint Surgery, 91(Suppl 2 Pt 1), 89–100. Available from: 10.2106/JBJS.H.01514 19255202

[16] Grood, E.S. , Stowers, S.F. & Noyes, F.R. (1988) Limits of movement in the human knee. Effect of sectioning the posterior cruciate ligament and posterolateral structures. The Journal of Bone & Joint Surgery, 70, 88–97. Available from: 10.2106/00004623-198870010-00014 3335577

[17] Guo, J. , Qi, C. , Zhang, D. , Yang, G. , Wang, C. , Yang, P. et al. (2023) Safe femoral tunnel drilling angles avoid injury to the medial and posteromedial femoral anatomic structures during single‐bundle posterior cruciate ligament reconstruction with the inside‐out technique. Knee Surgery, Sports Traumatology, Arthroscopy, 31, 3390–3398. Available from: 10.1007/s00167-023-07412-0 37039872

[18] Guth, J.J. , Brophy, R.H. , Matava, M.J. , Steinmetz, R.G. & Smith, M.V. (2022) Stress radiography is a reliable method to quantify posterior cruciate ligament insufficiency: a systematic review. Arthroscopy, Sports Medicine, and Rehabilitation, 4, e1851–e1860. Available from: 10.1016/j.asmr.2022.05.013 36312726 PMC9596873

[19] Harris, A.H.S. , Fernandes‐Taylor, S. & Giori, N. (2012) Not statistically different” does not necessarily mean “the same”: the important but underappreciated distinction between difference and equivalence studies. Journal of Bone and Joint Surgery, 94, e29. Available form: 10.2106/JBJS.K.00568 22398743

[20] Herbort, M. , Tecklenburg, K. , Zantop, T. , Raschke, M.J. , Hoser, C. , Schulze, M. et al. (2013) Single‐bundle anterior cruciate ligament reconstruction: a biomechanical cadaveric study of a rectangular quadriceps and bone—patellar tendon—bone graft configuration versus a round hamstring graft. Arthroscopy: The Journal of Arthroscopic & Related Surgery, 29, 1981–1990. Available from: 10.1016/j.arthro.2013.08.030 24140140

[21] Hughston, J.C. & Norwood, Jr., L.A. (1980) The posterolateral drawer test and external rotational recurvatum test for posterolateral rotatory instability of the knee. Clinical Orthopaedics and Related Research, 147, 82–87. Available from: 10.1097/00003086-198003000-00014 7371321

[22] Jiang, D. , Ao, Y. , Jiao, C. , Guo, Q. , Xie, X. , Zhao, F. et al. (2019) The effect of cyclic knee motion on the elongation of four‐strand hamstring autograft in anterior cruciate ligament reconstruction: an in‐situ pilot study. BMC Musculoskeletal Disorders, 20, 321. Available from: 10.1186/s12891-019-2699-5 31288779 PMC6615292

[23] Kato, T. , Śmigielski, R. , Ge, Y. , Zdanowicz, U. , Ciszek, B. & Ochi, M. (2018) Posterior cruciate ligament is twisted and flat structure: new prospective on anatomical morphology. Knee Surgery, Sports Traumatology, Arthroscopy, 26, 31–39. Available from: 10.1007/s00167-017-4634-3 28712026

[24] Kennedy, N.I. , Wijdicks, C.A. , Goldsmith, M.T. , Michalski, M.P. , Devitt, B.M. , Årøen, A. et al. (2013) Kinematic analysis of the posterior cruciate ligament, part 1: the individual and collective function of the anterolateral and posteromedial bundles. The American Journal of Sports Medicine, 41, 2828–2838. Available from: 10.1177/0363546513504287 24064797

[25] Krott, N.L. , Wengle, L. , Whelan, D. , Wild, M. & Betsch, M. (2022) Single and double bundle posterior cruciate ligament reconstruction yield comparable clinical and functional outcomes: a systematic review and meta‐analysis. Knee Surgery, Sports Traumatology, Arthroscopy, 30, 2388–2399. Available from: 10.1007/s00167-022-06907-6 35174403

[26] Makris, C.A. , Georgoulis, A.D. , Papageorgiou, C.D. , Moebius, U.G. & Soucacos, P.N. (2000) Posterior cruciate ligament architecture: evaluation under microsurgical dissection. Arthroscopy: The Journal of Arthroscopic & Related Surgery, 16, 627–632. Available from: 10.1053/jars.2000.9238 10976124

[27] Mannor, D.A. , Shearn, J.T. , Grood, E.S. , Noyes, F.R. & Levy, M.S. (2000) Two‐bundle posterior cruciate ligament reconstruction. An in vitro analysis of graft placement and tension. The American Journal of Sports Medicine, 28, 833–845. Available from: 10.1177/03635465000280061101 11101106

[28] Martin, R.B. , Burr, D.B. , Sharkey, N.A. & Fyhrie, D.P. (1998) Skeletal tissue mechanics, 190. New York, USA: Springer. Available from: 10.1007/978-1-4757-2968-9

[29] Merican, A.M. , Ghosh, K.M. , Deehan, D.J. & Amis, A.A. (2009) The transpatellar approach for the knee in the laboratory. Journal of Orthopaedic Research, 27, 330–334. Available from: 10.1002/jor.20755 18846554

[30] Michel, P.A. , Domnick, C. , Raschke, M.J. , Kittl, C. , Glasbrenner, J. , Deitermann, L. et al. (2019) Soft tissue fixation strategies of human quadriceps tendon grafts: a biomechanical study. Arthroscopy: The Journal of Arthroscopic & Related Surgery, 35, 3069–3076. Available from: 10.1016/j.arthro.2019.05.025 31405619

[31] Mouton, C. , Theisen, D. , Meyer, T. , Agostinis, H. , Nührenbörger, C. , Pape, D. et al. (2015) Combined anterior and rotational knee laxity measurements improve the diagnosis of anterior cruciate ligament injuries. Knee Surgery, Sports Traumatology, Arthroscopy, 23, 2859–2867. Available from: 10.1007/s00167-015-3757-7 26318487

[32] Oakes, D.A. , Markolf, K.L. , McWilliams, J. , Young, C.R. & McAllister, D.R. (2002) Biomechanical comparison of tibial inlay and tibial tunnel techniques for reconstruction of the posterior cruciate ligament. Analysis of graft forces. The Journal of Bone and Joint Surgery‐American Volume, 84, 938–944. Available from: 10.2106/00004623-200206000-00007 12063327

[33] Peez, C. , Deichsel, A. , Zderic, I. , Richards, R.G. , Gueorguiev, B. , Kittl, C. et al. (2024) Valgus malalignment causes increased forces on a medial collateral ligament reconstruction under dynamic valgus loading: a biomechanical study. Knee Surgery, Sports Traumatology, Arthroscopy, 32, 864–871. Available from: 10.1002/ksa.12110 38454816

[34] Petersen, W. & Zantop, T. (2010) Die arthroskopische Ersatzplastik des anterolateralen Bündels des hinteren Kreuzbandes in Einzelbündeltechnik mit autologer Semitendinosus‐/Grazilissehne. Operative Orthopädie und Traumatologie, 22, 354–372. Available from: 10.1007/s00064-010-9034-5 20931316

[35] Race, A. & Amis, A.A. (1996) Loading of the two bundles of the posterior cruciate ligament: an analysis of bundle function in a‐P drawer. Journal of Biomechanics, 29, 873–879. Available from: 10.1016/0021-9290(95)00161-1 8809617

[36] Race, A. & Amis, A.A. (1998) PCL reconstruction. In vitro biomechanical comparison of ‘isometric’ versus single and double‐bundled ‘anatomic’ grafts. The Journal of Bone and Joint Surgery. British Volume, 80, 173–179. Available from: 10.1302/0301-620X.80B1.0800173 9460977

[37] Ritchie, J.R. , Bergfeld, J.A. , Kambic, H. & Manning, T. (1998) Isolated sectioning of the medial and posteromedial capsular ligaments in the posterior cruciate ligament‐deficient knee. The American Journal of Sports Medicine, 26, 389–394. Available from: 10.1177/03635465980260030801 9617401

[38] Robinson, J.R. , Bull, A.M.J. , de W. Thomas, R.R. & Amis, A.A. (2006) The role of the medial collateral ligament and posteromedial capsule in controlling knee laxity. The American Journal of Sports Medicine, 34, 1815–1823. Available from: 10.1177/0363546506289433 16816148

[39] Schroven, W. , Vles, G. , Verhaegen, J. , Roussot, M. , Bellemans, J. & Konan, S. (2022) Operative management of isolated posterior cruciate ligament injuries improves stability and reduces the incidence of secondary osteoarthritis: a systematic review. Knee Surgery, Sports Traumatology, Arthroscopy, 30, 1733–1743. Available from: 10.1007/s00167-021-06723-4 34505176

[40] Strobel, M. , Weiler, A. , Konopatzki, H. , Gagstatter, F. & Telger, T.C. (2010) The posterior cruciate ligament: anatomy, evaluation, operative technique. Tuttlingen, Germany: Endo‐Press.

[41] Wijdicks, C.A. , Kennedy, N.I. , Goldsmith, M.T. , Devitt, B.M. , Michalski, M.P. , Årøen, A. et al. (2013) Kinematic analysis of the posterior cruciate ligament, part 2: a comparison of anatomic single‐ versus double‐bundle reconstruction. The American Journal of Sports Medicine, 41, 2839–2848. Available from: 10.1177/0363546513504384 24092043

[42] Willinger, L. , Runer, A. , Vieider, R. , Muench, L.N. , Siebenlist, S. & Winkler, P.W. (2024) Noninvasive and reliable quantification of anteromedial rotatory knee laxity: a pilot study on healthy individuals. The American Journal of Sports Medicine, 52, 1229–1237. Available from: 10.1177/03635465241234263 38506950 PMC10986148

[43] Winkler, P.W. , Zsidai, B. , Wagala, N.N. , Hughes, J.D. , Horvath, A. , Senorski, E.H. et al. (2021) Evolving evidence in the treatment of primary and recurrent posterior cruciate ligament injuries, part 1: anatomy, biomechanics and diagnostics. Knee Surgery, Sports Traumatology, Arthroscopy, 29, 672–681. Available from: 10.1007/s00167-020-06357-y PMC7917041 33201271

[44] Winkler, P.W. , Zsidai, B. , Wagala, N.N. , Hughes, J.D. , Horvath, A. , Senorski, E.H. et al. (2021) Evolving evidence in the treatment of primary and recurrent posterior cruciate ligament injuries, part 2: surgical techniques, outcomes and rehabilitation. Knee Surgery, Sports Traumatology, Arthroscopy, 29, 682–693. Available from: 10.1007/s00167-020-06337-2 PMC7917042 33125531

[45] Woo, S.L.Y. , Debski, R.E. , Withrow, J.D. & Janaushek, M.A. (1999) Biomechanics of knee ligaments. The American Journal of Sports Medicine, 27, 533–543. Available from: 10.1177/03635465990270042301 10424228

[46] Xiao, Y. , Liang, Z. , Shen, S. , Liu, F. , Hu, H. & Chen, B. (2023) Increased ACL direct insertion coverage provided more positive biomechanical effects on graft and bone tunnel during knee flexion: a simulation study. Journal of Experimental Orthopaedics, 10, 108. Available from: 10.1186/s40634-023-00677-x 37897510 PMC10613193

[47] Zhao, F. , Hu, X. , Zhang, J. , Shi, W. , Ren, B. , Huang, H. et al. (2019) A more flattened bone tunnel has a positive effect on tendon‐bone healing in the early period after ACL reconstruction. Knee Surgery, Sports Traumatology, Arthroscopy, 27, 3543–3551. Available from: 10.1007/s00167-019-05420-7 30877317

